# Empirical evidence for a process-based model of health-related quality of life using network analysis

**DOI:** 10.3389/fpubh.2025.1522133

**Published:** 2025-01-21

**Authors:** Nicolette Stogios, Troy Francis, Rachel G. Peiris, Aleksandra Stanimirovic, Valeria Rac, Robert P. Nolan

**Affiliations:** ^1^Campbell Family Mental Health Research Institute, Centre for Addiction and Mental Health, Toronto, ON, Canada; ^2^Cardiac eHealth, Peter Munk Cardiac Centre, University Health Network, Toronto, ON, Canada; ^3^Program for Health System and Technology Evaluation, Ted Rogers Centre for Heart Research, Toronto General Hospital Research Institute, University Health Network, Toronto, ON, Canada; ^4^Institute of Health Policy, Management and Evaluation, Dalla Lana School of Public Health, University of Toronto, Toronto, ON, Canada; ^5^Institute for Mental Health Policy Research, Centre for Addiction and Mental Health, Toronto, ON, Canada; ^6^Department of Psychiatry, University of Toronto, Toronto, ON, Canada

**Keywords:** health-related quality of life, network analysis, patient-reported outcomes, process-based approach, chronic heart failure, chronic kidney disease

## Abstract

**Background:**

Health-related quality of life (HRQL) is the perceived impact of a medical condition on one's overall wellbeing. While contemporary assessments are structured to evaluate an individual's HRQL state, we propose a complementary process-based model, which is defined as an appraisal that evolves over time as it reflects and informs a self-regulatory process of adapting to dynamic changes in bio-psycho-social life domains. In support of this approach, we developed a novel HRQL assessment tool called the EUROIA: **E**val**U**ation of goal-di**R**ected activities to pr**O**mote wellbe**I**ng and he**A**lth, which uses self-report data to assess the frequency with which individuals engage in a sample of goal-directed activities in pursuit of living well.

**Methods:**

We conducted a network analysis to evaluate the hypothesis that the EUROIA subscales would demonstrate a meaningful pattern of associations with an established HRQL measure and associated indices of psychosocial functioning and efficacy in self-managing a chronic medical condition.

**Results:**

The EUROIA is associated with established indices of HRQL in a manner that is theoretically consistent with our process-based model. Stability coefficients (i.e., betweenness, closeness, and strength) of the analysis revealed high reliability for the network.

**Conclusion:**

This analysis provides support for the validation of a process-based approach to HRQL assessment, which is represented, in part, by the EUROIA. A process-based approach complements and expands conventional measures of HRQL by focusing on how a patient's capacity to engage in goal-directed activities for living well is affected by their medical condition.

## Introduction

Health-related quality of life (HRQL) is defined as the perceived impact of a medical condition or therapy on physical, psychological, or social domains of one's wellbeing, assessed via patient-reported outcome measures (1). Patients living with chronic illnesses often exhibit impaired HRQL, which is assessed using single ratings (2) and multidimensional profiles (3, 4). There is an extensive range of HRQL instruments, with demonstrated validity and reliability, which serve as prognostic indicators for clinical outcomes (5, 6). Accordingly, policy statements advocate the assessment of HRQL as a primary endpoint in the evaluation of the effects of disease progression or the benefit of clinical interventions (7).

Previous work by our team identified three distinct models of wellbeing that are embedded in current HRQL assessments: (a) eudaimonic wellbeing [i.e., the perception of flourishing in personal growth and happiness as sampled by self-ratings for happiness or purpose in life (8, 9)], (b) hedonic wellbeing [i.e., the absence of symptoms of physical discomfort or emotional distress and the presence of elevated positive feelings and life satisfaction (10, 11)], and (c) desire-satisfaction [i.e., a state marked by fulfillment vs. frustration in attaining objects or experiences to which we are attracted (12, 13)]. Therefore, it is common to observe that individual HRQL assessments use an eclectic mix of items that reflect multiple dimensions of wellbeing across bio-psycho-social life domains. At the same time, these assessments are not necessarily designed to evaluate how an individual's self-reported HRQL state compares with a theoretically coherent model of wellbeing.

### The process-based model for living well

**Figure 1:**
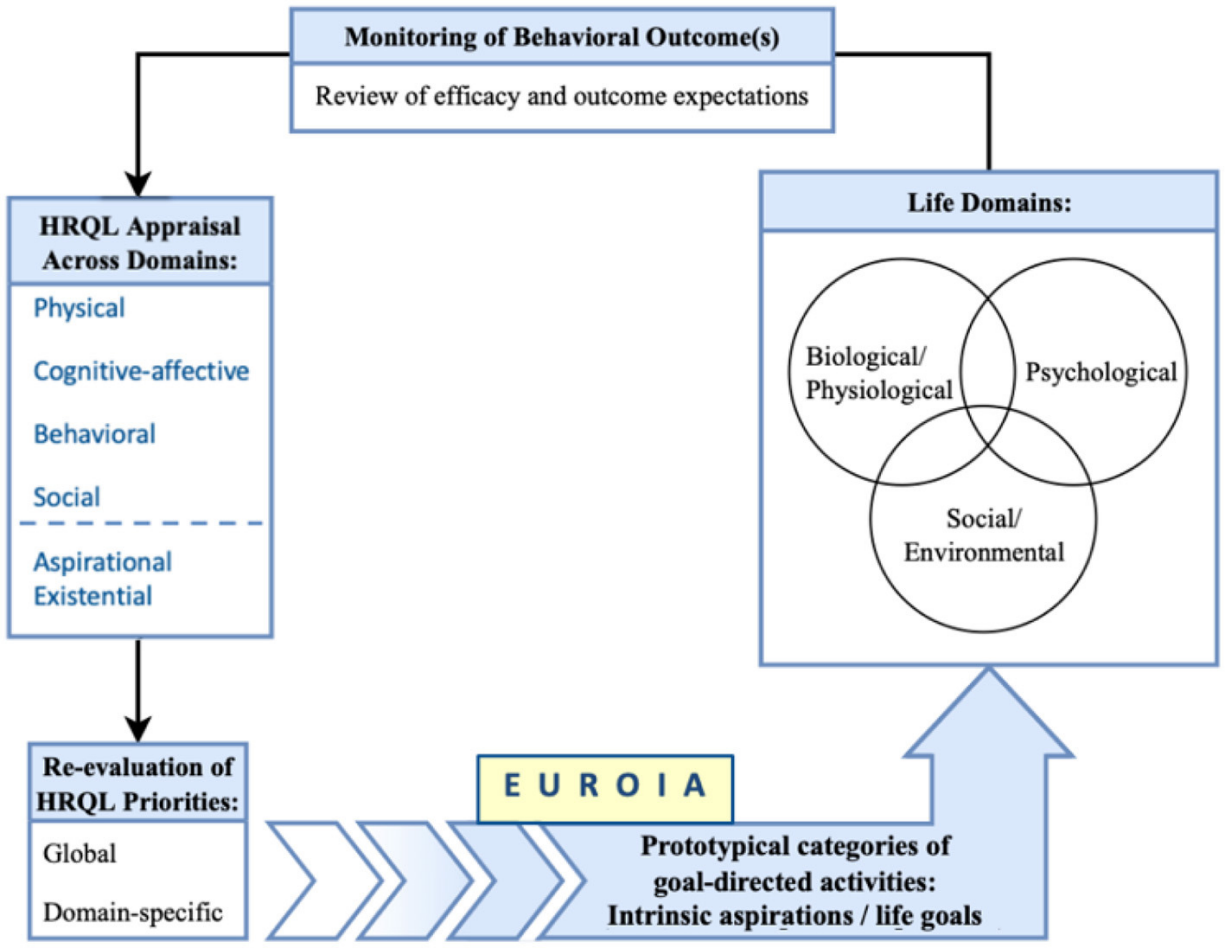
A process-based approach to HRQL as a meta-theoretical model.

Contemporary research on HRQL has aimed to operationally define the critical features of the state of wellbeing. There have been recent initiatives where a network analysis has strengthened this approach by specifying how a state of wellbeing emerges from a complex network of biopsychosocial variables (14). In contrast to a state based approach that aims to evaluate an individual's HRQL state, Nolan and Sharpe (59) introduced a process-based model which is an appraisal that evolves over time as it reflects and informs a self-regulatory process of adapting to dynamic changes in health status (14–17). Figure 1 illustrates key features of the process-based model of HRQL. Briefly, HRQL appraisals express the perceived impact of an illness or clinical treatment on one's wellbeing at a given point in time. These appraisals are associated with new or re-newed priorities for living well, which are expressed in terms of life goals or aspirations (17). In turn, one's aspirations evoke goal-directed activities that affect changes in bio-psycho-social life domains. Performance-based feedback from these activities is reviewed in terms of efficacy appraisals and outcome expectations. These then influence our re-appraisal of HRQL and re-evaluation of HRQL priorities. This iterative process is self-regulatory in nature. We adapt to life events across bio-pscyho-social domains while appraising our progress in fulfilling our aspirations for living well, which informs our recalibration of HRQL priorities and engagement in subsequent goal-directed activities.

The conceptualization of a process-based approach is based on four considerations. First, qualitative research with individuals diagnosed with a chronic progressive medical condition has shown that self-reported HRQL is part of a dynamic process of adapting to acute changes in their health status (18, 19). These changes are often unanticipated or recurrent, which can, in cases of progressive illness, evoke a sense of existential threat that challenges the individual to learn how to accept a life that feels fundamentally tenuous.

Second, current assessments evaluate an individual's HRQL profile against a state of idealized wellbeing where ratings for items are anchored by optimal levels of personal flourishing (eudaimonia), pleasure and satisfaction (hedonia), or fulfillment of desire. It is challenging to see how these ideals for wellbeing provide a meaningful standard of comparison for evaluating HRQL in individuals with pathophysiologic conditions that involve chronic impairment or suffering (e.g., chronic pain), or progressive functional limitations with premature morbidity and mortality (e.g., heart failure). Instead, the process-based approach to HRQL utilizes a concept from ancient Stoic philosophy in which the goal of attaining eudaimonic wellbeing (i.e., personal flourishing, mastery, or excellence) was reconceptualized by the goal of learning to live well with adversities or positive events in daily life —i.e., “good flow of life” (*euroia biou*). HRQL was presented as a process of responding to life events in a manner that affirmed our personal agency and dignity, as well as our ability to connect with our environment (20).

Third, systematic reviews and meta-analyses have established that a response shift is commonly observed in the way that individuals respond over time to HRQL assessments. This shift is expressed as (i) recalibrating how self-rating scales are used when gauging the severity of a given symptom or function, (ii) reprioritizing different features of HRQL to better reflect new insights or experiences about living well that have become personally salient, or (iii) reconceptualizing their understanding of the HRQL construct to reflect their evolving priorities for living well (21). This evidence indicates that the HRQL construct may be routinely undergoing change in its content and structure, which suggests that it may reflect an ongoing adaptive process.

The last point of consideration is the structure of the wellbeing construct that is embedded in HRQL assessments. From an empirical and theoretical standpoint, there is consensus that wellbeing is a multi-dimensional construct. At the same time, there are diverse accounts of its hierarchical structure, even though the core components are essentially the same across studies. The components usually include hedonic, eudaimonic, and social indices of wellbeing (22–24). In more recent years, proposals have been made for empirically integrating these separate models into a single unified structure (25, 26). Gallagher et al. (27) asserts that wellbeing is integrated within a primary hierarchical structure that contains three second-order latent factors of hedonic, eudaimonic and social dimensions of wellbeing (27). Other studies have also reported a primary hierarchical structure, though the specific components of wellbeing have varied. Bjørndal et al. proposed a structure where six distinct factors of wellbeing loaded onto a single higher-order factor that may represent a general index of happiness (28). On the other hand, Linton et al. reported that the dimensions of wellbeing clustered around 6 core themes, but without a primary hierarchical model (29). Similarly, Ruggeri et al. reported that 10 dimensions of wellbeing identified in their population survey could not be meaningfully aggregated into a single composite index of happiness or life-satisfaction, although they acknowledged that composite scores can be useful for summarizing change over time and for capturing its variation in social circumstances on a macro-level (30). Finally, van Woerkom et al. conducted a network analysis which showed that the nodes (or areas of interaction in their communities of variables) did not passively reflect a higher order causal agent of wellbeing or HRQL. Rather, the nodes were viewed as active agents in a causal system in which wellbeing was an emergent property of the interactions (31).

Given the diversity of state-based models for the structure of wellbeing that have emerged in the literature, two main interpretations of the findings are possible. Either only one of the proposed models on the structure of wellbeing/HRQL is correct, or each may present a profile that is valid. The latter case would suggest that the structure of HRQL/wellbeing is not fixed, and rather that it is dynamic and changes over time. We suggest that the reported structure of wellbeing would be expected to fluctuate over time according to an individual's ongoing adaptation to dynamic changes in bio-psycho-social domains of their personal environment. Arguably, it is a priority to evaluate the potential validity of this account.

Our team recently introduced a novel HRQL assessment, the EUROIA: **E**val**U**ation of goal-di**R**ected activities to pr**O**mote well-be**I**ng and he**A**lth. This scale represents a multi-dimensional profile of prototypical categories of goal-directed activities for living well (32–34). The scale uses self-report data to assess the frequency with which individuals engage in a sample of goal-directed activities that are identified with their pursuit of living well (wellbeing). These goal-directed activities represent a key feature of our process-based model of HRQL. Psychometric properties of the EUROIA were previously described in terms of reliability, content validity, and clinical utility using various methodological approaches including Exploratory Principal Axis Factor (ePAF) analyses and Confirmatory Factor Analysis (33, 34). In this study, we aimed to evaluate the integrity and credibility of the overall process-based model of HRQL. We conducted a network analysis to evaluate whether EUROIA subscales would demonstrate a meaningful pattern of associations with established HRQL measures and with associated indices of psychosocial functioning and efficacy in self-managing a chronic medical condition. Thus, this work is innovative and has the potential to advance the field of wellbeing/HRQL by introducing a new model that complements conventional state-based measures of HRQL. Our pursuit of living well is defined as a self-regulatory process that occurs as part of a complex adaptive system as we adapt to, and interact with dynamic changes in the biopsychosocial domains of our lived experience.

## Methods

### Participants and data sources

This investigation was a sub-study of the Open Access Digital Community Promoting Self-Care, Peer Support, and Health Literacy—A Virtual Community Promoting Mental Health, Psychosocial Adjustment, and Peer Support (ODYSSEE-vCHAT) projects: a single group, open label, pre-post study (35) and a double-arm, parallel group, randomized controlled trial (36). The ODYSSEE-vCHAT (35) study recruited participants aged 18 years and older with a proficiency in English and a diagnosis of chronic heart failure (CHF; NYHA Class II to IV for at least 3 months prior to enrollment) or advanced chronic kidney disease (CKD; < 10% risk of requiring dialysis within 2 years or end-stage renal disease and receiving dialysis). Complete study details can be found elsewhere (35). The present network analysis was based on complete case data from the baseline assessments of the ODYSSEE-vCHAT study and trial. Only participants with complete data on the relevant variables were included in the analysis. Any participants with missing data were excluded.

The sample estimate for the ODYSSEE-vCHAT study accounted for changes in the Mental Component Summary (MCS) of the Short Form 36 (SF-36) Health Survey (37) over a period of 3 to 12 months, while the sample estimate for the ODYSSEE-vCHAT trial was based on change over 12 months in a composite index of all-cause mortality and hospitalization. The trial was estimated to have a sample size *of N* = 162, while the study had a sample size *of N* = 188, which included oversampling. Both the study and trial were adjusted for a potential 12-month withdrawal or attrition rate of 14.7%, a type 1 error of 5%, and a power of 80% (38).

### Measures

The EUROIA measure includes dimensions related to eudaimonic wellbeing (EUD-WB), self-affirmation (SLF-AFF), social affiliation (SOC-AFF), and social roles and responsibilities (SOC-RR). *Eudaimonia* is composed of behaviors that promote flourishing and self-actualization aimed at personal growth, connection to a greater purpose, and life satisfaction (39, 40). *SOC-AFF* is comprised of behaviors such as participation in social activities, maintaining close relationships, and helping others. *SOC-RR* assess behaviors related to maintaining roles and obligations to significant others and being productive in a work-related setting. *SLF-AFF* focuses on behaviors related to physical activity and exercise and includes activities that promote feelings of being healthy and attractive and maintaining positive affect. Established indices of HRQL used to validate the EUROIA subscales are summarized in Table 1.

**Table 1:** HRQL scales used in network analysis.

**Measure of HRQL**	**Description**
Short Form 36 (SF-36) Health Survey Mental Component Summary (MCS) (37)	A questionnaire of 36 items that assess patients' health status and its impact on their lives. Consists of various multi-item scales: Role Limitation due to Emotional Problems (RE), Emotional Well-Being (EWB), Vitality (VT), and Social Functioning (SF).
Revised UCLA Loneliness Scale (RULS-6) (54)	A short version of the 20-item scale measuring subjective feelings of loneliness and social isolation.
Flourishing Scale (FS) (56)	An 8-item summary measure of self-perceived success in areas such as relationships, self-esteem, purpose, and optimism.
Self-Efficacy for Managing Chronic Diseases 6-item Scale (SEMCD-6) (65)	A 6-item scale that covers several common domains across many chronic diseases, including symptom control, role function, emotional functioning and communicating with physicians.
ENRICHD Social Support Index (ESSI) (55)	A 7-item self-report questionnaire to assess social support.
Godin-Shephard Leisure-Time Physical Activity Questionnaire (GSLTPAQ) (53)	A 4-item questionnaire used to assess leisure-time physical activity (any leisurely activity undertaken by the individual that increases their total energy expenditure).

### Network analysis

Network analysis was used to complement and extend the results of the EUROIA and its association with established HRQL indices, as summarized in Table 1. Graphical least absolute shrinkage and selection operator (glasso) were performed to estimate the network structure of the EUROIA and HRQL indices using the extended Bayesian information criterion (EBIC). A polychloric correlation matrix was computed, which provided the foundation of the network. This allowed for the examination of partial correlations between each subscale while controlling for all the other variables in the network (41). We constructed a network wherein each subscale was represented as a node and the partial correlations between the items as edges. Networks were displayed using a Fruchterman-Reingold algorithm (42) whereby nodes with stronger connections are placed at the center of the network and weaker connections more peripherally. Analyses were performed using R software v4.1.0 (43), qgraph v1.9.8 (44), glasso v1.11 (45), bootnet v1.6 (46), psych v2.4.3 (47), and igraph v2.0.2 (48).

### Network centrality measures

Centrality refers to several metrics that determine a node's relative importance compared to other nodes in the network (49). Strength centrality reflects a node being central through having strong connections to other nodes based on the absolute sum of the weighted number and strength of its connections relative to all other nodes. Betweenness centrality determines how important a node is in connecting other nodes by identifying the frequency with which a node lies on the shortest path between two other nodes. Closeness centrality measures the distance between nodes and quantifies the node's relationship to all other nodes in the network (50). Bootstrapping with 2,500 permutations was done to assess the stability of the centrality metrics. The generated stability coefficients demonstrated values >0.5, indicating high reliability, while values of 0.25 indicated the minimum evidence. We used the Correlation Stability coefficient (CS-coefficient) for correlation values equal to or above r = 0.7 to measure the stability of centrality indices (46). The CS-coefficient indicates the percentage of our sample that can be dropped to maintain, with a 95% confidence interval, correlation values equal to or above r = 0.7 between our sample's centrality indices and our bootstrapped samples' centrality indices. Non-parametric bootstrap (resampling rows with replacement) was employed to create 1,000 samples to estimate edge weight stability (46).

### Network sub-community identification

Network sub-communities or clusters were identified through exploratory graph analysis (EGA), which uses random walk algorithms to identify dimensions in psychometric data (51). Clusters seek to identify areas in the network with nodes have many connections within, and few connections between, clusters (41). The walktrap method was selected as communities identified using this method are shown to be consistent with latent factors of factor models (51). Item stability statistics were calculated to evaluate the strength of the placement of each item within the derived dimensions. The proportion of times that each item is placed in each dimension was calculated to estimate item stability. This calculation is useful in identifying which items contribute to the consistency of the structure of the dimensions by frequently replicating in the same dimension, and which items lead to inconsistency by frequently replicating in other dimensions. Variables at or below the range of 0.65 to 0.75 are considered to be less stable (52).

## Results

A total of 276 participants with complete baseline data were included in the network analysis. Patients included in the sample had a primary diagnosis of either CHF (50.5%) or CKD (49.5%). The mean age was 56.6 years (range: 20.0 to 91.0, SD = 16.0) and 59.6% were male. The majority of the sample (65.0%) had completed a college or university degree and another 23.0% completed graduate or post-graduate degrees. Respondents were primarily identified as having a White or Caucasian racial background (58.2%), living with a partner (59.1%), and had a combined household income ≥ $100 000 CAD.

### Network analysis

**Figure 2:**
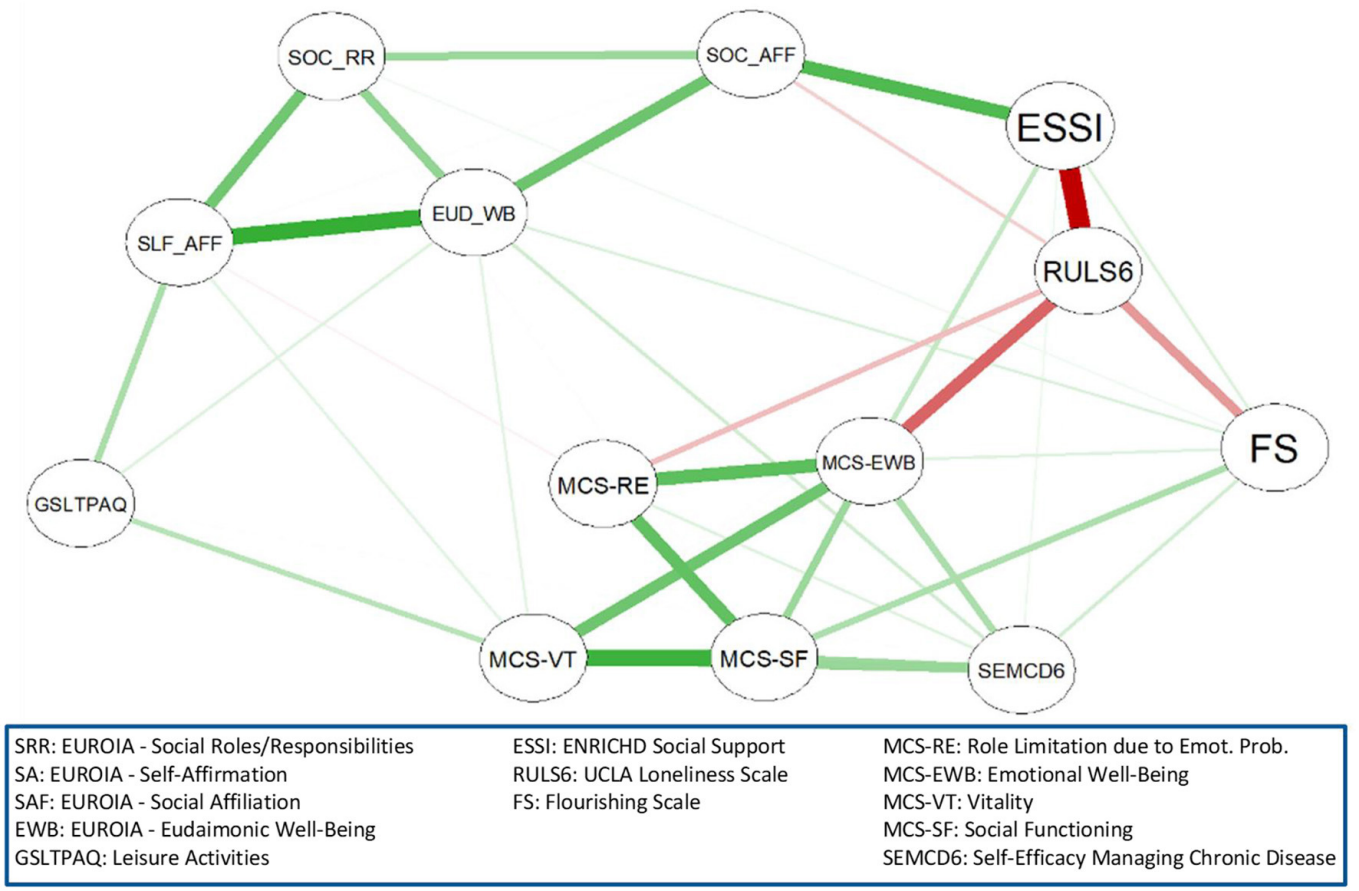
Network structure of the EUROIA and associated HRQL indices.

**Figure 3:**
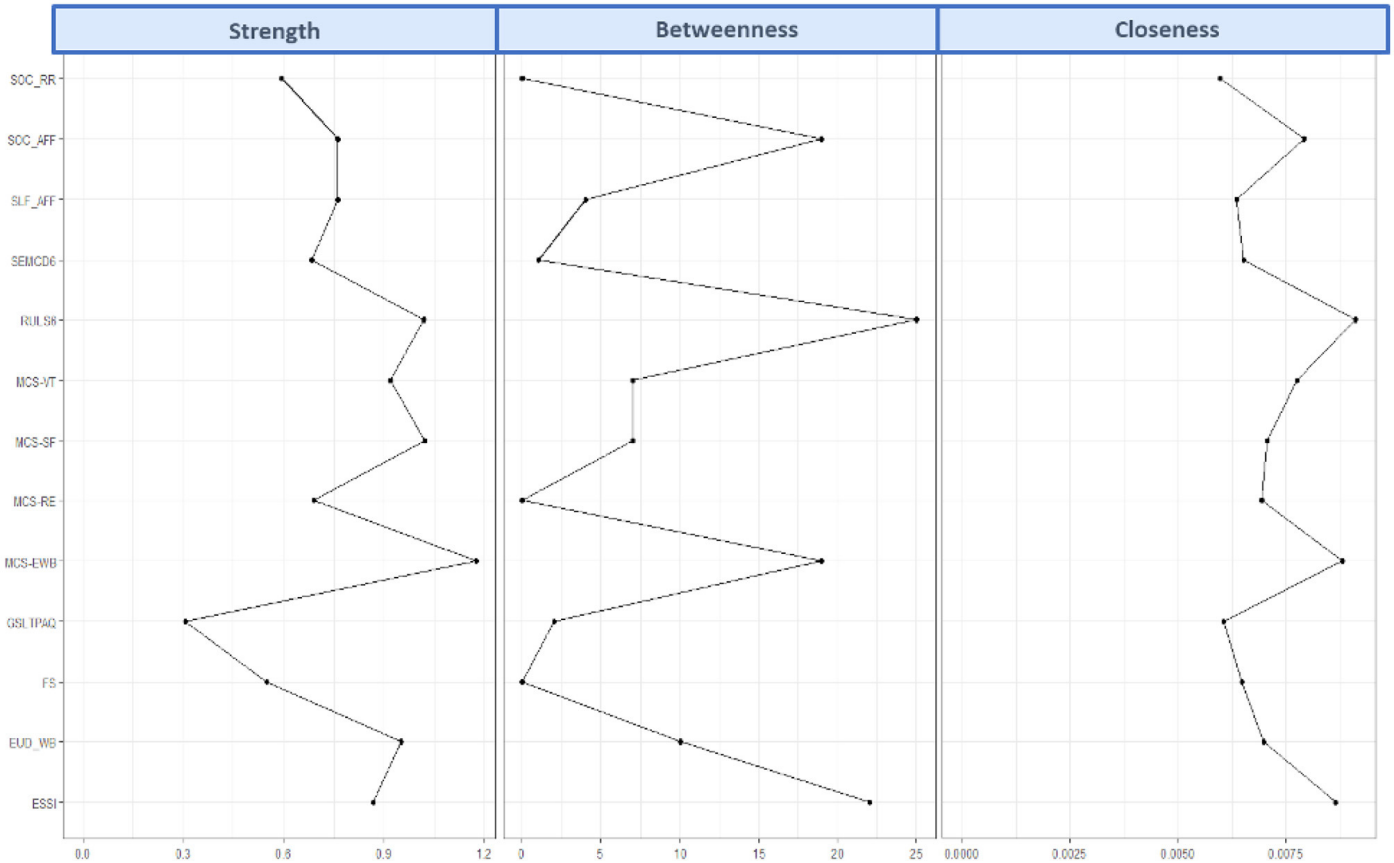
Strength centrality, closeness centrality, and betweenness centrality of each EUROIA subscale.

The glasso network of the EUROIA and HRQL indices is shown in Figure 2. The network was comprised of 13 nodes, 40 edges (31 positive and 9 negative) out of a possible 78 connections, a mean edge weight of 0.039, and a network density of 0.51 (51% of nodes were directly connected). A green connection indicates a positive link while red connections signify negative links. The width (thickness) of the connection depicts the strength of the partial correlations. Network centrality values are presented in Figure 3 and Supplementary Table S1. MCS-Emotional Wellbeing (EWB) was shown to be the most influential node in the network, with a strength centrality of 1.64. The Godin-Shephard Leisure-Time Physical Activity Questionnaire (GSLTPAQ) (53) demonstrated the weakest centrality (−2.08). The Revised UCLA Loneliness Scale (RULS-6) (54) (1.75) and ENRICHD Social Support Index (ESSI) (55) (1.42) exhibited the highest betweenness centralities and were shown to mediate the connections between other nodes, primarily the EUROIA and MCS subscales. Additionally, the RULS-6, MCS-EWB, and ESSI were the most closely connected subscales. The EUROIA subscales were linked to MCS subscales via positive connections with social support (ESSI) and psychological flourishing [Flourishing Scale (FS) (56)], and through an inverse association with loneliness (RULS-6). Additional pathways to the MCS involved self-efficacy and leisure activity (GSLTPAQ).

### Network accuracy and stability

The tests for network accuracy and stability are presented in the Supplementary material S2, S3. Bootstrapping was used to compute robust centrality estimates represented as correlation stability coefficients. The correlation stability coefficients were 0.28 (ranging from 0.21 to 0.36) for betweenness, 0.44 (ranging from 0.36 to 0.52) for closeness, and 0.67 (ranging from 0.59 to 0.75) for strength. The minimum 0.25 cut-off was met, indicating that the centrality metrics can be interpreted with confidence and the network can be considered reliable (46).

### Community detection

**Figure 4:**
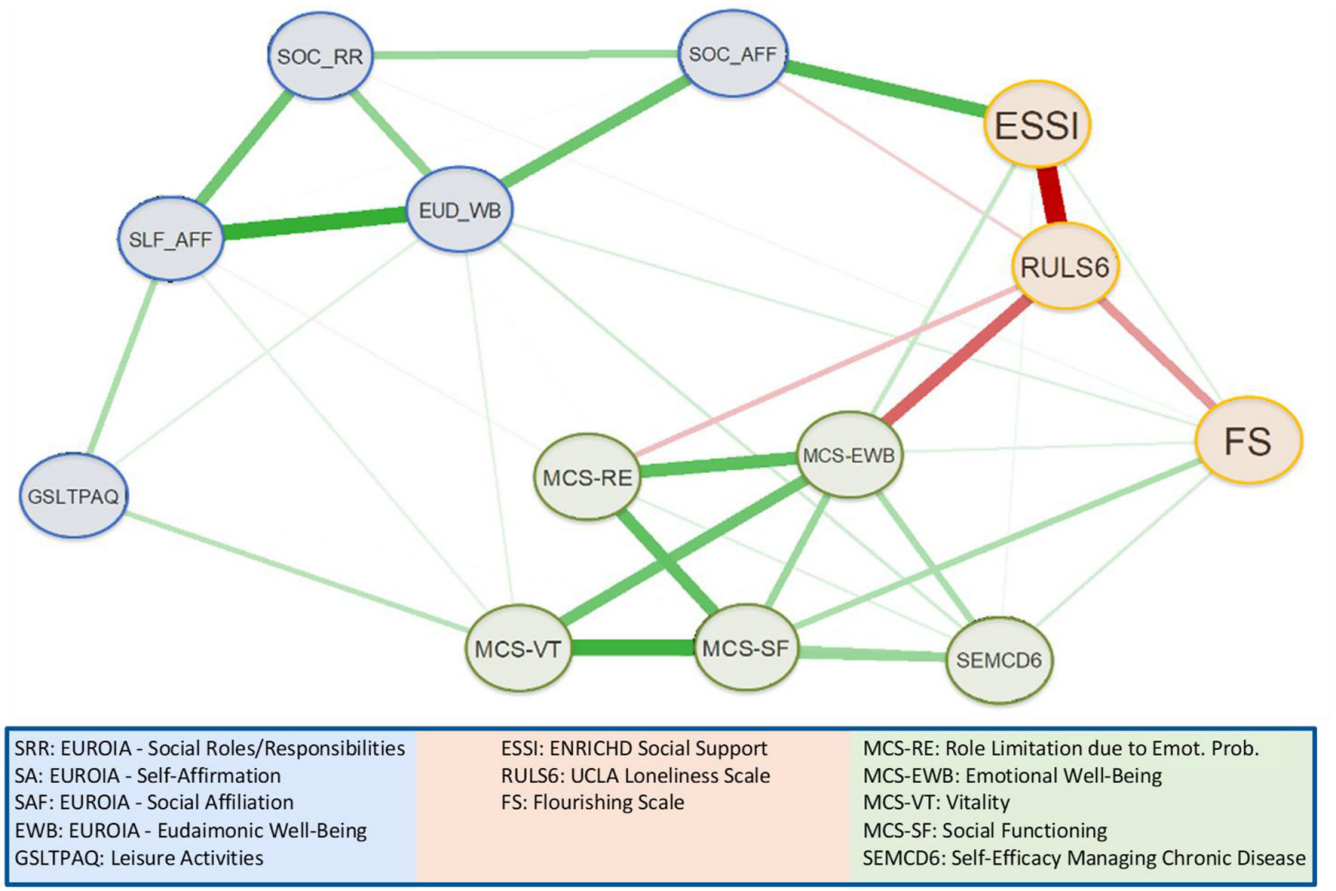
HRQL network sub-community identified using exploratory graph analysis.

Figure 4 shows the glasso network map with the EGA sub-communities highlighted. Notably, three sub-communities were identified, of which two matched *a priori* theme groupings reflecting the EUROIA scale (SOC-AFF, SOC-RR, EUD-WB, SLF-AFF) and the MCS Role Limitation due to Emotional Problems (RE), Emotional Wellbeing (EWB), Vitality (VT), and Social Functioning (SF) subscales (37). The themes identified EUROIA and leisure activities, MCS and self-efficacy, and social support, loneliness, and flourishing. Item stability statistics (Supplementary Figure S4) for each dimension were close to 1, demonstrating the robustness of the communities estimated using EGA.

## Discussion

In this empirical study, we present a process-based, meta-theoretical model of HRQL that complements established measures of an HRQL state, while also introducing a novel dynamic feature of this construct. Using network analysis, we demonstrate that the EUROIA, an assessment of goal-directed activities for living well, is associated with established indices of HRQL in a manner that is theoretically consistent with our process-based model. More specifically, EUROIA subscales were linked to components of HRQL as measured by MCS subscales, via social support, psychological flourishing, and through an inverse association with loneliness. Additional pathways to the MCS involved self-efficacy and leisure activity. Importantly, stability coefficients (i.e., betweenness, closeness, and strength) of the analysis revealed high reliability for the network.

### Meta-theoretical underpinnings of a process-based approach to HRQL

The process-based approach presented by Nolan and Sharpe (Figure 1) is best defined as a meta-theoretical model informed by relevant theories of behavior change, including Self-Determination Theory (17), Motivational Interviewing (57), the Transtheortical Model of Health Behavior Change (58), and the social-cognitive theory of agentic change (16). Our model posits the following:

- HRQL is an appraisal that evolves as it reflects our effort to adapt to dynamic changes in health status (59) and to challenges or facilitating factors in our biopsychosocial environment.
- HRQL appraisals are associated with new or revised aspirations that are personally salient and meaningful (60, 61).
- These aspirations subsequently inform and evoke goal-directed activities for living well, which are expressed in our self-regulated or self-determined effort to promote change associated with improved wellbeing (14–17).
- Performance-based feedback from these goal-directed activities is reviewed and expressed as efficacy and outcome expectations (16); efficacy expectations are organized along a continuum that ranges from intrinsic to extrinsic sources of motivation/causality, and this locus of causality influences the degree to which a goal-directed activity for living well may be sustained (17).
- The evaluation of outcomes from our goal-direced activities, in turn, influences our re-appraisal of HRQL, which carries forward in this complex system to re-shape subsequent aspirations and goal-directed actions that ultimately promote our adaptation across bio-psycho-social life domains.

The EUROIA is an instrument that evaluates a key component of a process-based model of HRQL/wellbeing and, as such, has the potential to contribute meaningfully to recent theoretical/philosophical and empirical initiatives in this area. For example, use of the EUROIA in applied research complements the call for “mid-level theories” of wellbeing, as expressed by Alexandrova (62). She has asserted that abstract (normative) theories of HRQL or wellbeing (e.g., eudaimonic and hedonic) are only obscurely associated with real-life sitatuations, and fail to promote our understanding of how a specific individual might appraise wellbeing in a given life circumstance. In keeping with a process-based model, the EUROIA offers a mid-level approach to identifying prototypical categories of goal-directed activities through which individuals pursue their aspirations for living well within the parameters of daily life. Both qualitative and quantitative studies of these self-reported goal-directed activities should provide novel information about how HRQL appraisals are *relativized* to an individual's daily life and their pursuit of wellbeing (59).

Furthermore, our use of the EUROIA within a process-based approach to HRQL and wellbeing builds on the concept of response shift that was coined by Sprangers and Schwartz (63). Their research highlights that individuals living with a chronic pathophysiologic condition or illness are inclined to change their internal standards, values, and personal conceptualization of HRQL (63). In response, it is challenging on theoretical grounds for state-based models of HRQL assessment to assimilate findings of response shift. Assessments used in that approach are premised on the theory that items, subscales, and summary scores depicting an individual's HRQL state are organized according to a fixed psychometric structure, and dynamic changes in the organization of an individual's self-reported HRQL profile are attributed to “noise” or error variance (64). Moreover, these assessments evaluate an individual's HRQL profile according to its departure from a score that represents an idealized state whereby one has abundant energy and emotional tranquility all or most of the time, there are no symptoms of emotional stress or physical discomfort, there is no limitation on activities of daily living or social or work activities, and one's health is viewed as being excellent at present and into the future: c.f. SF-36 (37). It is unclear how this clinical standard for evaluating HRQL or wellbeing is relevant to the common experience of individuals who are interacting with natural challenges and demands of life that naturally evolve over time.

### Empirical evidence from the EUROIA to support a process-based approach to HRQL

**Figure 5:**
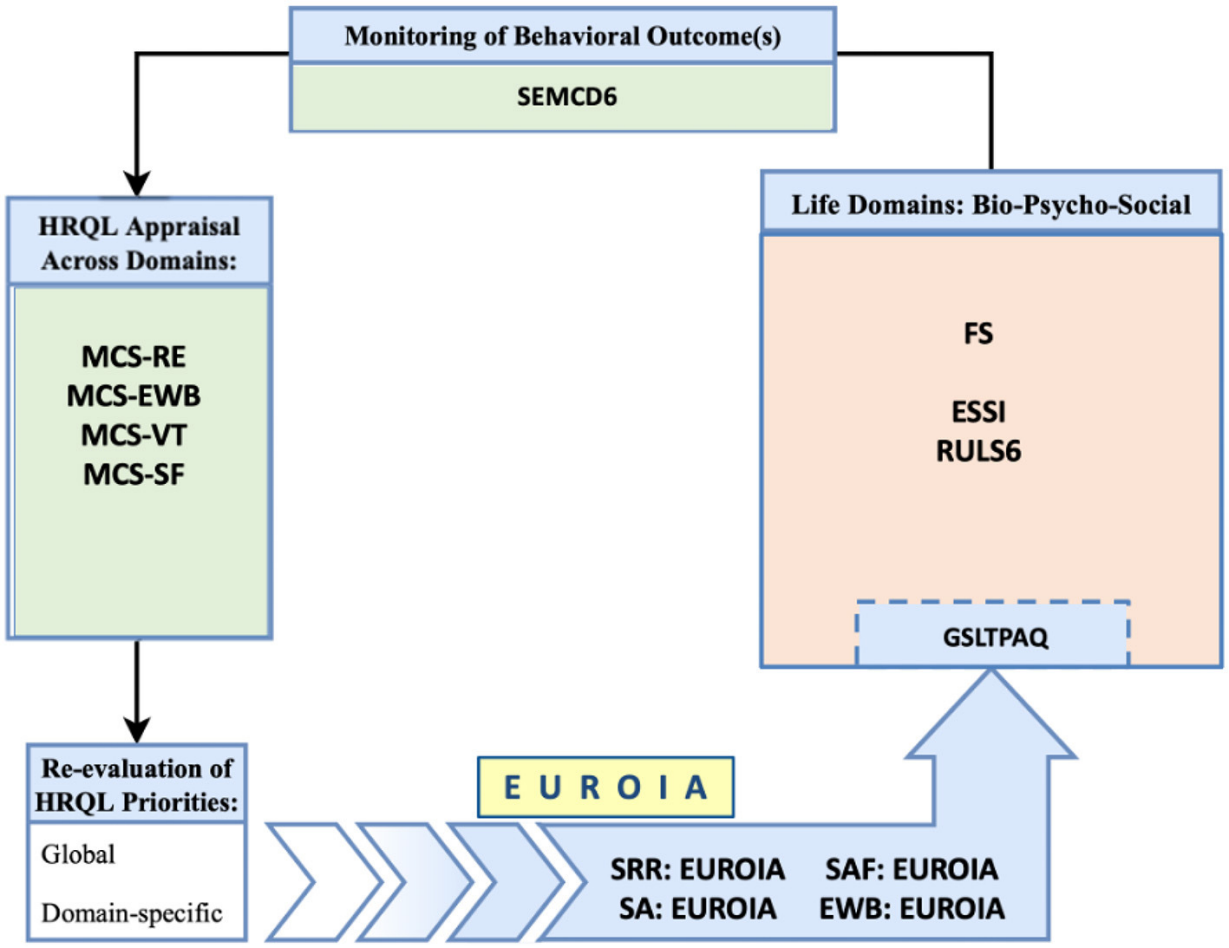
Correlation of sub-communities identified in network analysis with the theoretical model.

The EUORIA provides a multi-dimensional profile of prototypical categories of goal-directed activities for living well that we have described previously (32–34). Categories that have emerged to date are identified by the following EUROIA subscales: eudaimonic wellbeing (EUD-WB), self-affirmation (SLF-AFF), social affiliation (SOC-AFF), and social roles and responsibilities (SOC-RR). The network analysis presented in this study demonstrated that the EUROIA subscales were associated with the MCS community of variables (RE, EWB, VT, SF subscales) via pathways that were consistent with our theoretical model (Figure 5) (37). Indices such as the GSLTAS (53), FS (56), ESSI (55), and RULS6 (54) reflect psychosocial interactions or stressors in one's bio-psycho-social environment, and the Self-Efficacy for Managing Chronic Diseases 6-item Scale (SEMCD-6) (65) reflects perceived efficacy in self-managing one's medical condition. The directionality of the associations between the EUROIA and the MCS via the intermediary communities of variables noted above was aligned with our expectations. Higher scores on the ESSI, FS, GSLTPAQ, and SEMCD-6 were associated with greater HRQL (53, 55, 66), while a higher score for loneliness (RULS6) was associated with decreased HRQL (67). These findings underscore the clinical implications of the EUROIA, i.e., its potential to be used by medical practitioners in conjunction with standards of practice to support the maintenance or improvement of patients' HRQL. Patients with chronic illness and multiple morbidities often experience a reduction in HRQL (69–72) that, in turn, is correlated with the severity of their health status (73, 74). However, researchers have found that the education and empowerment of patients regarding HRQL activities can have lasting positive effects on self-reported levels of HRQL. The EUROIA, therefore, offersis a tool an opportunity forthat can be used by clinicians to work collaboratively partner with patients to address their concerns about health outcomes from a more holistic perspective.

### Methodological considerations and limitations

This study utilized network analysis to re-examine data and variables that were previously identified using latent variable methods (41, 60, 68). The subcommunities we identified for the EUROIA closely match the latent variables identified in Francis et al. (34), which provides confirmatory evidence for its general underlying structure. Nevertheless, there remain a number of limitations to the present study. Our use of network analysis was exploratory and should be treated with caution as the connections between nodes cannot be treated as true causal relationships and may be impacted by unobserved factors. Our subset of data was cross-sectional and does not reflect the dynamic nature of the networks. To improve our confidence in the underlying structure of the EUROIA and our process-based approach to HRQL, this study must be replicated using longitudinal time series analyses with an adequate sample size. Additionally, the generalizability of our findings may be limited due to the relatively high socioeconomic status of our sample. A future aim would be to modify the EUROIA scale to better incorporate aspects that could improve its applicability to a wider and more diverse cross-section of the population, taking into consideration sociocultural and demographic influences on the prototypical activities people engage in to live well. Despite these limitations, we believe this study makes a strong contribution to the credence of our meta-theoretical model of HRQL/wellbeing. It and demonstrates the applicability importance of the EUROIA scale to address a critical feature of this model in identifying the prototypical goal-directed activities one engages in to pursue their goals of for living well that promote subjective wellbeing/HRQL through a complex network of interations. It should also be noted that our previous work with the EUROIA evaluated the personal meaning and salience of each self-reported activity for living well by means of a separate subscale assessment. A priority for future studies with the EUROIA is to re-introduce this subscale where each item is self-rated according to both its frequency (F), perceived priority/importance (P), and F^*^P cross-product. In addition, a priority for future studies with the EUROIA will be to employ sampling methods that are more effective in recuitinga more socioeconomically representative sample.

## Conclusions

This study presents a process-based model of HRQL that highlights the dynamic nature of this construct. Use of the EUROIA in applied research represents a mid-level theoretical strategy that contributes to current efforts to clarify how HRQL appraisals are applied in daily efforts to maintain or improve our personal wellbeing. Moreover, the EUROIA introduces a preliminary summary of prototypical categories of goal-directed activities associated with HRQL/wellbeing. The process-based approach to HRQL assessment, which is partially represented by the EUROIA, complements and extends conventional measures by addressing how this patient-reported appraisal fits within a complex system of self-determined or self-regulated adaptation to one's medical condition.

## Data Availability

The raw data supporting the conclusions of this article will be made available by the authors, without undue reservation.
