# Supplementary material for: Empirical evidence for a process-based model of health-related quality of life using network analysis
Source: Front Public Health. 2025 Jan 21;13:1522133. doi: 10.3389/fpubh.2025.1522133 (PMC11790454; doi:10.3389/fpubh.2025.1522133)
Supplement: Supplementary file 1 [file Data_Sheet_1.pdf]

Empirical Evidence for a Meta-Theoretical Process-Based Model of Health-Related Quality of Life Using Network Analysis

Supplemental Materials

Table S1: Correlation Matrix for HRQOL Scales 2

Table S2: Raw Centrality Indices 3

Figure S1: Results from edge weight accuracy 4

Figure S2: Results from node centrality stability tests 5

Figure S3: Item stability statistics 6

Table S1: Correlation Matrix for HRQOL Scales

Variable	EUD_WB	SLF_AFF	SOC_AFF	SOC_RR	MCS-VT	MCS-SF	MCS-RE	MCS-EWB	RULS6	SEMCD6	ESSI	GSLTPAQ	FS
**EUD_WB**	1	0.5162	0.4484	0.4291	0.2709	0.2132	0.102	0.2161	-0.1855	0.2647	0.1937	0.2241	0.2444
**SLF_AFF**	0.5162	1	0.2899	0.4281	0.2213	0.0591	-0.0054	0.1035	-0.0735	0.1818	0.1534	0.269	0.1506
**SOC_AFF**	0.4484	0.2899	1	0.3653	0.1626	0.1658	0.1396	0.1855	-0.4106	0.1693	0.508	0.0829	0.2527
**SOC_RR**	0.4291	0.4281	0.3653	1	0.0855	0.0093	0.0099	0.0644	-0.0949	0.0553	0.1373	0.1309	0.1738
**MCS-VT**	0.2709	0.2213	0.1626	0.0855	1	0.6299	0.4421	0.6024	-0.4056	0.5034	0.2561	0.2505	0.249
**MCS-SF**	0.2132	0.0591	0.1658	0.0093	0.6299	1	0.5752	0.617	-0.4315	0.5136	0.307	0.1474	0.4034
**MCS-RE**	0.102	-0.0054	0.1396	0.0099	0.4421	0.5752	1	0.6091	-0.4654	0.4069	0.3004	0.0267	0.2262
**MCS-EWB**	0.2161	0.1035	0.1855	0.0644	0.6024	0.617	0.6091	1	-0.6145	0.5094	0.4961	0.143	0.409
**RULS6**	-0.1855	-0.0735	-0.4106	-0.0949	-0.4056	-0.4315	-0.4654	-0.6145	1	-0.3673	-0.6709	-0.0621	-0.4437
**SEMCD6**	0.2647	0.1818	0.1693	0.0553	0.5034	0.5136	0.4069	0.5094	-0.3673	1	0.3214	0.1698	0.3349
**ESSI**	0.1937	0.1534	0.508	0.1373	0.2561	0.307	0.3004	0.4961	-0.6709	0.3214	1	0.0217	0.3654
**GSLTPAQ**	0.2241	0.269	0.0829	0.1309	0.2505	0.1474	0.0267	0.143	-0.0621	0.1698	0.0217	1	0.1026
**FS**	0.2444	0.1506	0.2527	0.1738	0.249	0.4034	0.2262	0.409	-0.4437	0.3349	0.3654	0.1026	1

Table S2: Raw Centrality Indices

Node	Strength	Betweenness	Closeness
EUROIA - Social Affiliation	-0.12	1.10	0.61
EUROIA - Self Affirmation	-0.13	-0.54	-0.86
EUROIA - Roles/Responsibilities	-0.85	-0.97	-1.22
EUROIA - Eudaimonic Well-being	0.67	0.12	-0.28
ENRICHD Social Support	0.33	1.42	1.29
Leisure Activities	-2.08	-0.75	-1.13
UCLA Loneliness Scale	0.97	1.75	1.72
Flourishing Scale	-1.04	-0.97	-0.76
MCS - Role Limitation	-0.44	-0.97	-0.32
MCS - Emotional Well-being	1.64	1.10	1.42
MCS - Vitality	0.54	-0.21	0.44
MCS - Social Functioning	0.98	-0.21	-0.19
Self-efficacy Managing Chronic Disease	-0.46	-0.86	-0.71

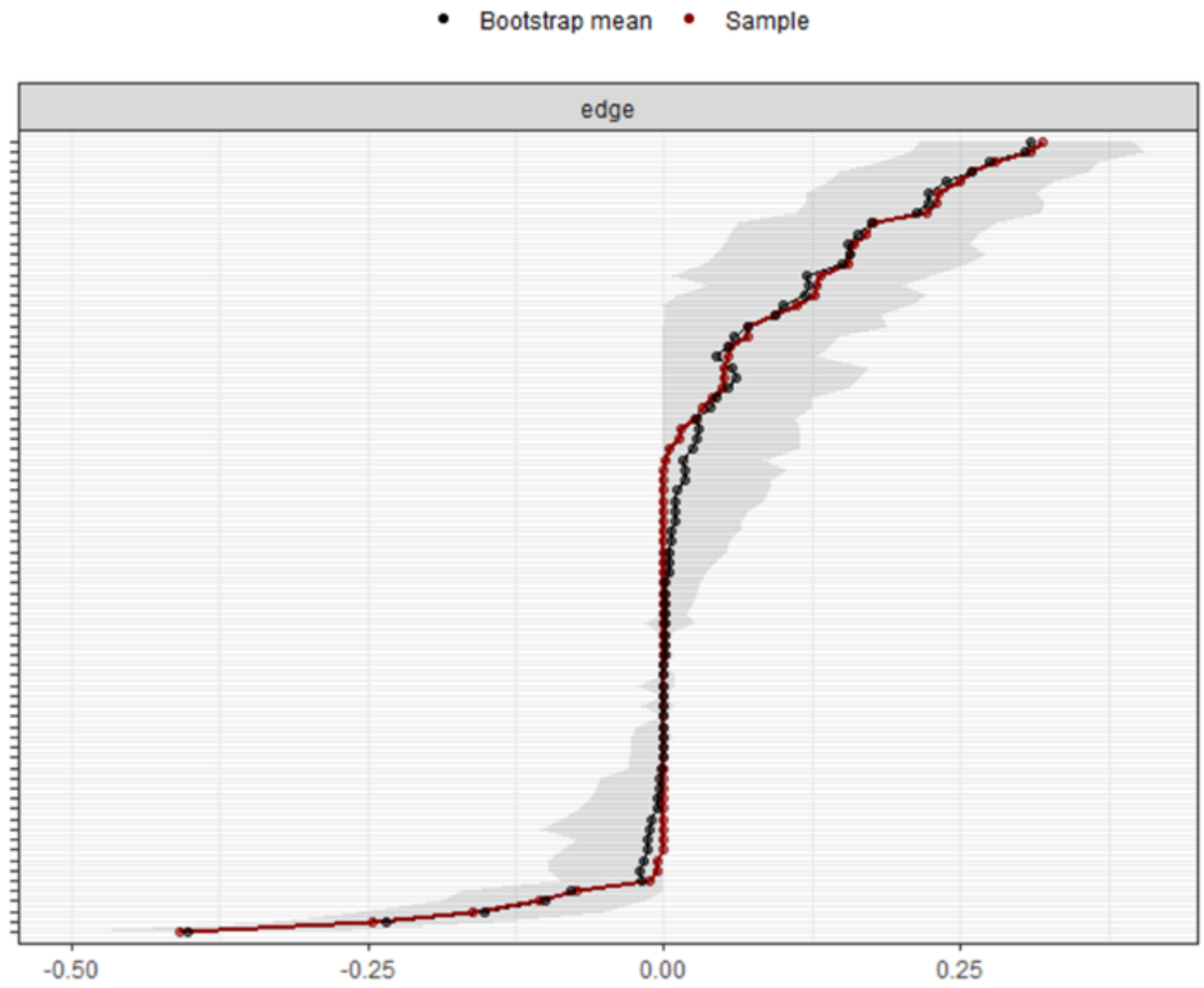

Figure S1: Results from edge weight accuracy

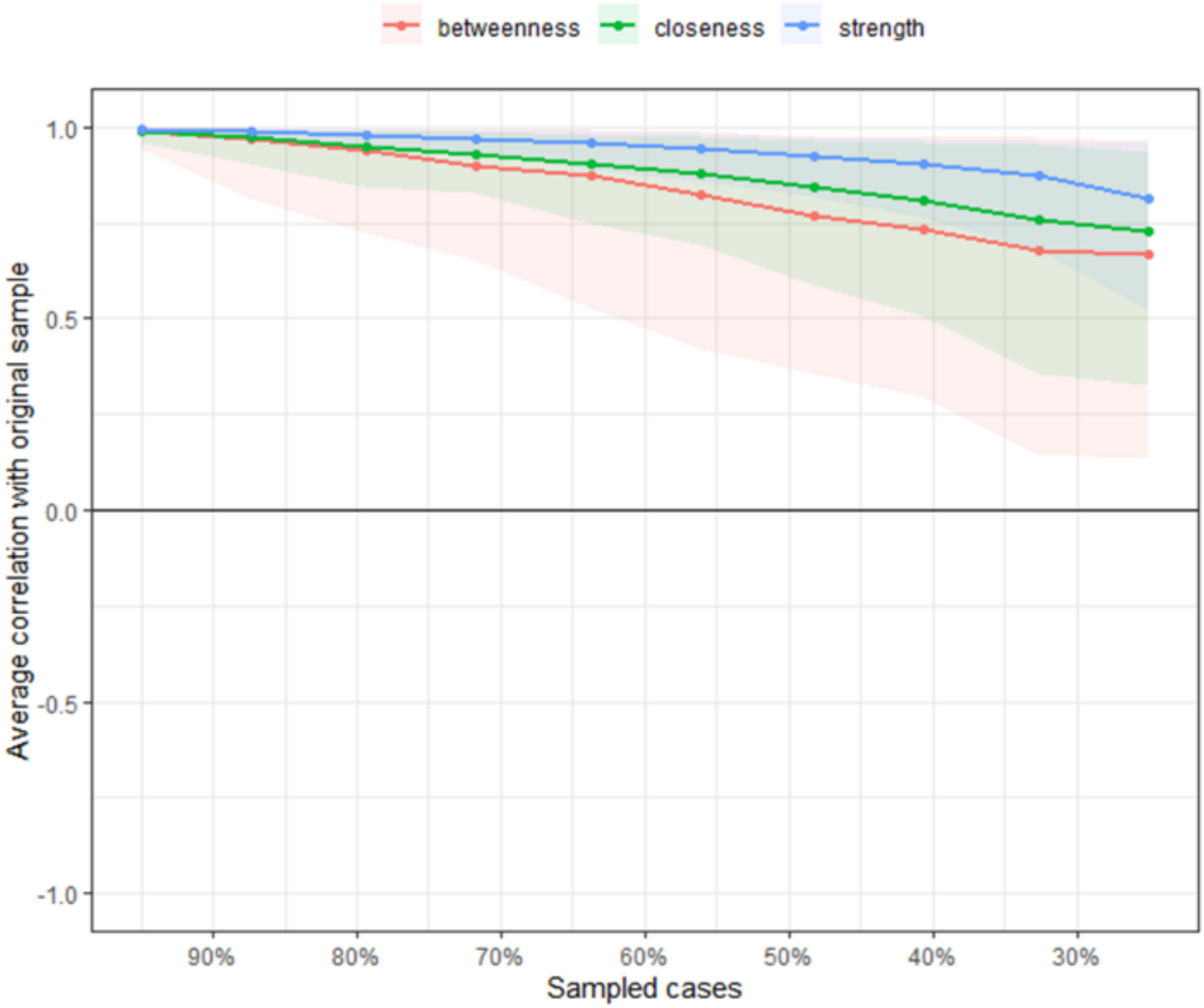

Figure S2: Results from node centrality stability tests

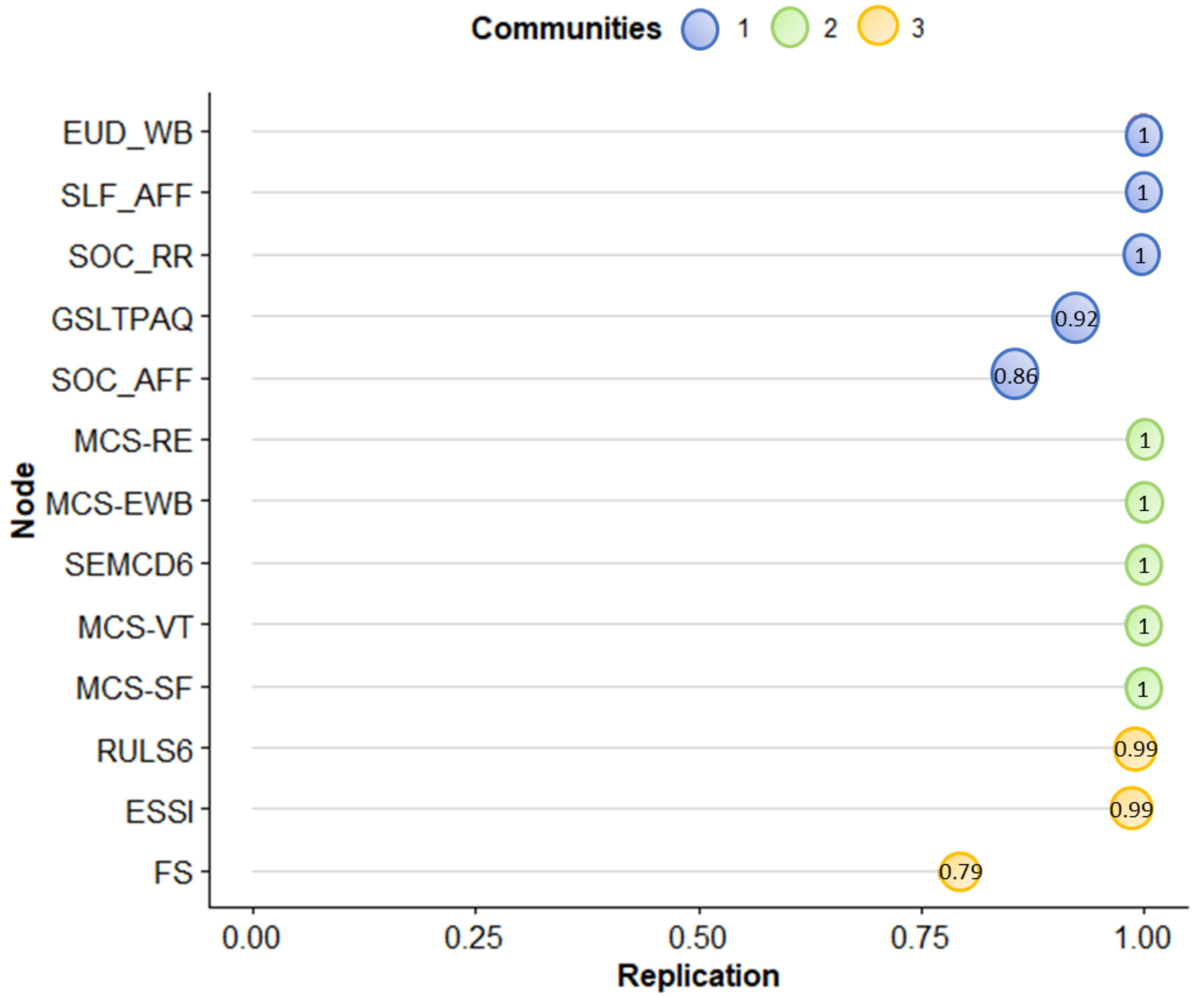

Figure S3: Item stability statistics
